# Meeting of the State Medical Society

**Published:** 1854-03

**Authors:** 


*Meeting of the State Medical Society.*—The society convened in its annual meeting, at the City Hall, in Albany, on the 7th of February, and continued in session till the afternoon of the 9th.

We were not present till the morning of the second day. We found a fair representation from the county societies and colleges, and a goodly number of permanent members. The attendance was somewhat larger than usual, owing, partly, to a better and more united feeling in the profession.

The proceedings were characterized by a good degree of harmony. Short speeches were the order of the day, except when the everliving subject of quackery was brought up. There was, we noticed, a general indisposition, on the part of the members, to stultify themselves by a long-winded discussion of empiricism. They seemed to think that it was as hopeless a task to abolish it, as to do away with original sin; and, finally, all resolutions offered were met with a cold reception, and either withdrawn or rejected—a sensible conclusion, we think, for yearly resolutions against quackery, are very like a yearly confession of faith for a church member. Once for all should answer.

The morning of the second day, after the disposition of these resolutions, was mainly passed in listening to a report sent by Dr. Willard Parker, through the hands of Dr. Alonzo Clark, upon Croup. It was an analytic record of a large number of cases in Dr. Parker's practice, which had been collected and arranged with much care by Dr. Thos. B. Cock.

The results arrived at were very curious, and, to our sense, would not be justified by a larger experience. But it is fair to state what were the conclusions, and to say that they were inevitable from the character of the cases.

1st. The operation of tracheotomy in croup, was found to be unsuccessful in so large a proportion of cases, that Dr. Parker considers it "unsurgical."

2d. The treatment by the topical application of nitrate of silver was found to be singularly unsuccessful.

3d. The treatment by large, and even enormous doses of calomel, at frequent intervals, arrayed better results in its favor than any other course of management.

4th. Combined with this was given, in almost every case, a lobelia mixture, of tincture of lobelia, syrup of ipecac, and some other nauseant which we have forgotten. With it, vaporized air was breathed by filling the room with steam.

In regard to the operation of tracheotomy, the unfavorable results obtained did not surprise us much, knowing, as we did, that it was never performed until it might almost be considered a *post-mortem.* It was mentioned by one member present, that in one case where he had made a post-mortem examination, he found the larynx only covered with false membrane, the trachea being healthy. Now, in this case, it was very evident that an operation would have insured the life of the child. Many other similar cases may occur without our knowledge. We can recall, at least, two cases of death by croup, where the dryness of the tracheal and bronchial sounds did not indicate false membrane in those localities.

But it is well known that many operations have been successful where there was false membrane in the trachea. The greatest constriction of the tube must ever be in the larynx, and it is a point gained to find entrance for air into the large and unelastic windpipe.

We are sorry, therefore, that Prof. Parker has thus censured this operation. Undoubtedly it must, in a large proportion of cases, be followed by death, but the true questions are:

First, does it increase the probability of death? and, secondly, does it afford an additional *chance* to the patient?

If we answer the first query in the negative, and the second in the affirmative, as it would seem we must do, then is the operation not "unsurgical," but called for at an earlier period than usually performed.

In regard to the nitrate of silver, we can only say that the few cases of recovery from diptheritic croup we have witnessed, or known, have been treated by the probang and caustic. A friend, in whose opinion we place much confidence, considers the sponge, and not the nitrate, as the efficient agent.

The calomel treatment, which was pursued in nearly all the successful cases, was also used, with equal energy, in those which died; and on this, as on all the other points discussed, there are conflicting opinions and testimony.

The discussion on croup was animated and able. When we have the Transactions, we shall take pains to give Dr. Parker's paper a careful examination. The high authority from which it springs, and the startling character of its results, call for a thoughtful insearch.

The afternoon was given to the election of officers for the ensuing year. A large number of votes were cast. Charles Brodhead Coventry, of Utica, was elected President upon the first ballot; a most excellent selection, and an honor worthily bestowed upon a deservedly eminent man. All the other offices were, so far as our knowledge goes, well filled.

Late in the afternoon, the Board of Censors appointed last year, at its previous meeting, to attend the examinations of the medical colleges in the city of New York, reported that the College of Physicians and Surgeons, in Crosby street, and the New York Medical College, in Fourteenth street, had invited and admitted them to their examinations of candidates for the degree of Doctor of Medicine. They reported that all the graduates of those schools were worthy of the degree.

From the University of the city of New York, in Thirteenth street, they reported that they had been refused the privilege or right of attending its examinations; that an answer from the faculty was deferred till a month after the time of graduation; and that, finally, a letter was received from Prof. Draper, assigning reasons for declining the supervision of the State society. The letter was read and embodied in the report.

On the third day the matter was called up. A very general expression of the opinions of members was given. The course of the college was considered derogatory to the society, and subversive of all restraints in the conferring of degrees. A resolution was unanimously passed, censuring the college in strong terms, as being desirous of concealing its operations from the inspection of an honorable profession.

The Board of Censors were again appointed, with instructions to hold no intercourse with the University school unless specially invited so to do.

A Committee of Inquiry was then appointed to inquire how it was that the Geneva Medical College had conferred an honorary degree upon an advertising quack. This was done at the request of members of the faculty then present, and (also at their request) a resolution was passed requesting boards of trustees to confer no more degrees without consulting their professors.

The society visited the Albany Medical College, and were well pleased with the department of practical chemistry. It adjourned at 3 o'clock, P. M., of the 9th.

Dr. Hun entertained the members at his house on the evening of the 7th, and Dr. Armbsy did the same, in a very pleasant style, on the evening of the 8th. H.

