# Correction

**Published:** 1854-05

**Authors:** 


### EDITORIAL DEPARTMENT.

*Correction.*— An error is noticed in our account of the proceedings of the N. Y. State Medical Society, relative to the visitation of the colleges by the State Censors. We inadvertently confounded the University of New York (under the Presidency of Prof. Draper) with the New York Medical College, in mentioning them, respectively, as the 13th st. and 14th st. schools.

The New York Medical College is in *Thirteenth st.*, and *did* invite the Censors to its examinations, as did also the Crosby st. school. The University of New York, on *Fourteenth st.*, refused to admit the Censors, on the ground that it was "a national school," and not under the jurisdiction of any state authority, though we believe it never hesitates to accept any donations of state moneys.

The error is merely in confusing the names of the streets. As it (though evidently unintentionally on our part) does injustice to the New York Medical College, we hope that those journals which have copied this portion of our report, will do us the justice to copy also our correction. H.

